# Cardiac tamponade due to pyopneumopericardium from malignant bronchopericardial fistula

**DOI:** 10.1007/s12471-017-0961-8

**Published:** 2017-03-27

**Authors:** T. M. Frisoli, T. Jain, T. Swadia, X. Hong, M. Guerrero

**Affiliations:** 1 Department of Cardiology, Henry Ford Hospital, Detroit, MI USA; 2 Department of Cardiology, Michigan Heart, St Joseph Mercy Health System, Livonia, MI USA; 3 Department of Radiology, Henry Ford Hospital, Detroit, MI USA; 4 Department of Cardiology, Evanston Hospital, North Shore University Health System, Evanston, IL USA

A 71-year-old female with lung adenocarcinoma underwent surveillance PET/CT, which revealed new central necrotic cavitation of a preexisting left lower lobe mass, with communication between this air-filled cavity and the left mainstem bronchus, as well as pericardial effusion with large pneumopericardium, consistent with malignant bronchopericardial fistula (Fig. 1). Echocardiography and physical examination confirmed tamponade. Bronchoscopic debulking showed a necrotic tumour cavity. During pericardiocentesis, air in the pericardial space was conspicuous fluoroscopically (Video 1). Intra-pericardial pressure was 11 mm Hg, 1500 ml of seropurulent fluid was removed. Fluid cultures were positive for *Staphylococcus aureus*. Her rest dyspnoea improved and she was discharged to hospice care.

**Fig. 1:**
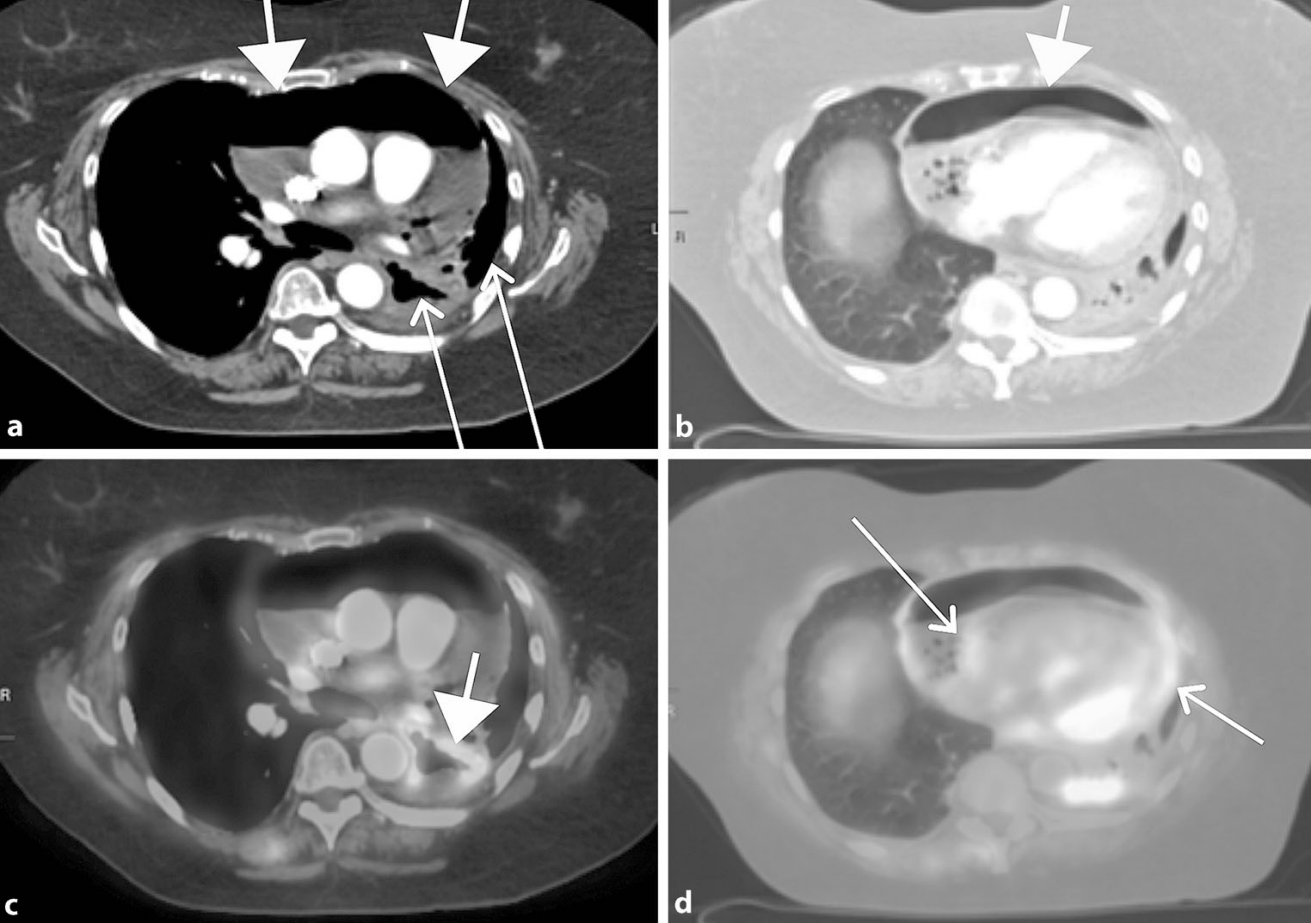
**a, b** CT demonstrates abnormal communication between the air-containing core of the necrotic lung mass with the left mainstem bronchus (*arrows*), as well as a large pneumopericardium (*wider arrowheads*) which is anterior to the heart. **c, d** PET CT demonstrates intense hypermetabolic air-containing cavitary large soft tissue mass in the left lower lobe (*wider arrowhead*), and moderate to intense hypermetabolic activity in the fibrous and parietal pericardium (*arrows*), which may represent infection and/or malignancy

Pneumopericardium is known to cause cardiac tamponade [1] in trauma patients or newborn infants requiring positive pressure ventilation. Fistulas such as between the oesophagus, stomach, or lung [2, 3] and pericardium have been reported. The unique images and video presented are illustrative of this interesting disease entity.

## Caption Electronic Supplementary Material

Video 1: Pneumopericardium as seen on fluoroscopy just prior to pericardiocentesis

## References

[1] Cummings RG, Wesly RL, Adams DH, Lowe JE (1984). Pneumopericardium resulting in cardiac tamponade. Ann Thorac Surg.

[2] George LD, David N, Omrani A, Davies R (1999). Bronchogenic carcinoma presenting as a bronchopericardial fistula. Int J Clin Pract.

[3] Harris RD, Kostiner AI (1975). Pneumopericardium associated with bronchogenic carcinoma. Chest.

